# Modulation of the Major Paths of Carbon in Photorespiratory Mutants of *Synechocystis*

**DOI:** 10.1371/journal.pone.0016278

**Published:** 2011-01-21

**Authors:** Jan Huege, Jan Goetze, Doreen Schwarz, Hermann Bauwe, Martin Hagemann, Joachim Kopka

**Affiliations:** 1 Department of Physiology and Cell Biology, Leibniz Institute of Plant Genetics and Crop Plant Research, Gatersleben, Germany; 2 Department of Chemistry, University of Potsdam, Potsdam-Golm, Germany; 3 Department of Plant Physiology, University of Rostock, Rostock, Germany; 4 Department of Molecular Physiology, Max-Planck-Institute of Molecular Plant Physiology, Potsdam-Golm, Germany; Queen Mary University of London, United Kingdom

## Abstract

**Background:**

Recent studies using transcript and metabolite profiles of wild-type and gene deletion mutants revealed that photorespiratory pathways are essential for the growth of *Synechocystis* sp. PCC 6803 under atmospheric conditions. Pool size changes of primary metabolites, such as glycine and glycolate, indicated a link to photorespiration.

**Methodology/Principal Findings:**

The ^13^C labelling kinetics of primary metabolites were analysed in photoautotrophically grown cultures of *Synechocystis* sp. PCC 6803 by gas chromatography-mass spectrometry (GC-MS) to demonstrate the link with photorespiration. Cells pre-acclimated to high CO_2_ (5%, HC) or limited CO_2_ (0.035%, LC) conditions were pulse-labelled under very high (2% w/w) ^13^C-NaHCO_3_ (VHC) conditions followed by treatment with ambient ^12^C at HC and LC conditions, respectively. The ^13^C enrichment, relative changes in pool size, and ^13^C flux of selected metabolites were evaluated. We demonstrate two major paths of CO_2_ assimilation via Rubisco in *Synechocystis*, i.e., from 3PGA via PEP to aspartate, malate and citrate or, to a lesser extent, from 3PGA via glucose-6-phosphate to sucrose. The results reveal evidence of carbon channelling from 3PGA to the PEP pool. Furthermore, ^13^C labelling of glycolate was observed under conditions thought to suppress photorespiration. Using the glycolate-accumulating *ΔglcD1* mutant, we demonstrate enhanced ^13^C partitioning into the glycolate pool under conditions favouring photorespiration and enhanced ^13^C partitioning into the glycine pool of the glycine-accumulating *ΔgcvT* mutant. Under LC conditions, the photorespiratory mutants *ΔglcD1* and *ΔgcvT* showed enhanced activity of the additional carbon-fixing PEP carboxylase pathway.

**Conclusions/Significance:**

With our approach of non-steady-state ^13^C labelling and analysis of metabolite pool sizes with respective ^13^C enrichments, we identify the use and modulation of major pathways of carbon assimilation in *Synechocystis* in the presence of high and low inorganic carbon supplies.

## Introduction

Cyanobacteria are considered the first organisms to have evolved the capacity for oxygenic photosynthesis around three billion years ago [1]. The endosymbiotic uptake of an ancient cyanobacterial ancestor by a eukaryotic cell initiated the evolution of phototrophic algae and plants. Many of the initial cyanobacterial proteins are still detectable within the chloroplasts and nuclear genomes of current higher plants [2], [3]. In both cyanobacteria and C3 plants, CO_2_ fixation is primarily catalysed by the enzyme ribulose-1,5-bisphosphate carboxylase/oxygenase (Rubisco). The carboxylase reaction generates two molecules of 3-phosphoglycerate (3PGA) from ribulose-1,5-bisphosphate and CO_2_, whereas O_2_ competition at the reaction centre leads to the oxygenase products 3PGA and 2-phosphoglycolate (2PG) [4]. The product 2PG is a cellular toxin that needs to be detoxified, as it inhibits Calvin-Cycle enzymes [5]–[7]. In plants, 2PG is scavenged by a sequence of reactions called the photorespiratory C2 pathway [8]–[10], which regenerates one molecule of 3PGA for every two molecules of 2PG, at the cost of CO_2_ and NH_4_^+^ release.

In contrast to higher plants, 2PG metabolism was thought to exert negligible effects in cyanobacteria. Early studies only indicated the formation of glycolate from 2PG [11]. Furthermore, the discovery of a sophisticated inorganic carbon (C_i_) concentration mechanism (CCM) demonstrated the potential of cyanobacteria to increase the internal concentration of CO_2_ in the vicinity of Rubisco and thus to compensate for the low CO_2_ affinity of the cyanobacterial enzyme [12]. As a consequence, the CCM was thought to be sufficient to suppress the oxygenase reaction and to make photorespiratory detoxification irrelevant for cyanobacterial metabolism.

Recent studies, however, demonstrated not only that the CCM is insufficient to prevent ribulose-1,5-bisphosphate oxygenation in an O_2_-containing atmosphere but also that there is active 2PG metabolism. The photorespiratory pathways were found to be essential for growth under atmospheric conditions [13], [14]. Photorespiratory 2PG metabolism in the *Synechocystis* sp. strain PCC 6803 (hereafter *Synechocystis*) comprises three alternative paths: a plant-like C2 cycle, a bacterial glycerate pathway, and the complete decarboxylation of glyoxylate via oxalate. Mutants defective in specific enzymes within these pathways including glycolate dehydrogenase (Δ*glcD1*, *sll*0404) and glycine decarboxylase (Δ*gcvT*, *sll*0171) displayed growth retardation. In addition, intermediates of photorespiratory 2PG metabolism accumulated in agreement with the enzymatic defect; these intermediates included glycolate in Δ*glcD1* and glycine in Δ*gcvT*. These phenomena were already apparent under elevated CO_2_ conditions (5%, high CO_2_, HC), and were enhanced under the low-CO_2_-availability conditions of ambient air (∼0.035%, low CO_2_, LC) [14], [15]. The C_i_ affinity and the maximum rate of photosynthesis were found to be higher in cells grown under LC conditions than in cells grown under HC conditions [14].

Transcript and metabolite profiles of the wild-type (WT) strain compared to the above-mentioned mutants indicated that the photorespiratory mutants, when grown under HC conditions, display gene expression and metabolite patterns that are characteristic for WT cells when shifted from HC to LC conditions [15], [16]. Characterisation of changes of metabolite pools opened the path towards currently unanswered questions, including: (1) Is the previously observed accumulation of intermediates, such as glycolate and glycine, indeed linked to photorespiration? (2) How is the ratio of Rubisco oxygenation to carboxylation influenced by altered C_i_ availability? (3) Which routes of carbon fixation are used under HC or LC conditions? To approach these questions, we applied a non-steady state ^13^C_i_ pulse and chase experimental design similar to work previously published by our lab [17]. The appearance and dilution of ^13^C labelled intermediates within the primary metabolism of photoautotrophically grown WT and selected mutant cells was monitored following the suggestions made for dynamic flux analyses [18]. With this approach, we reveal the use and modulation of the major paths of carbon assimilation in *Synechocystis*.

## Results

### Experimental design of metabolic flux analysis

**Figure 1:**
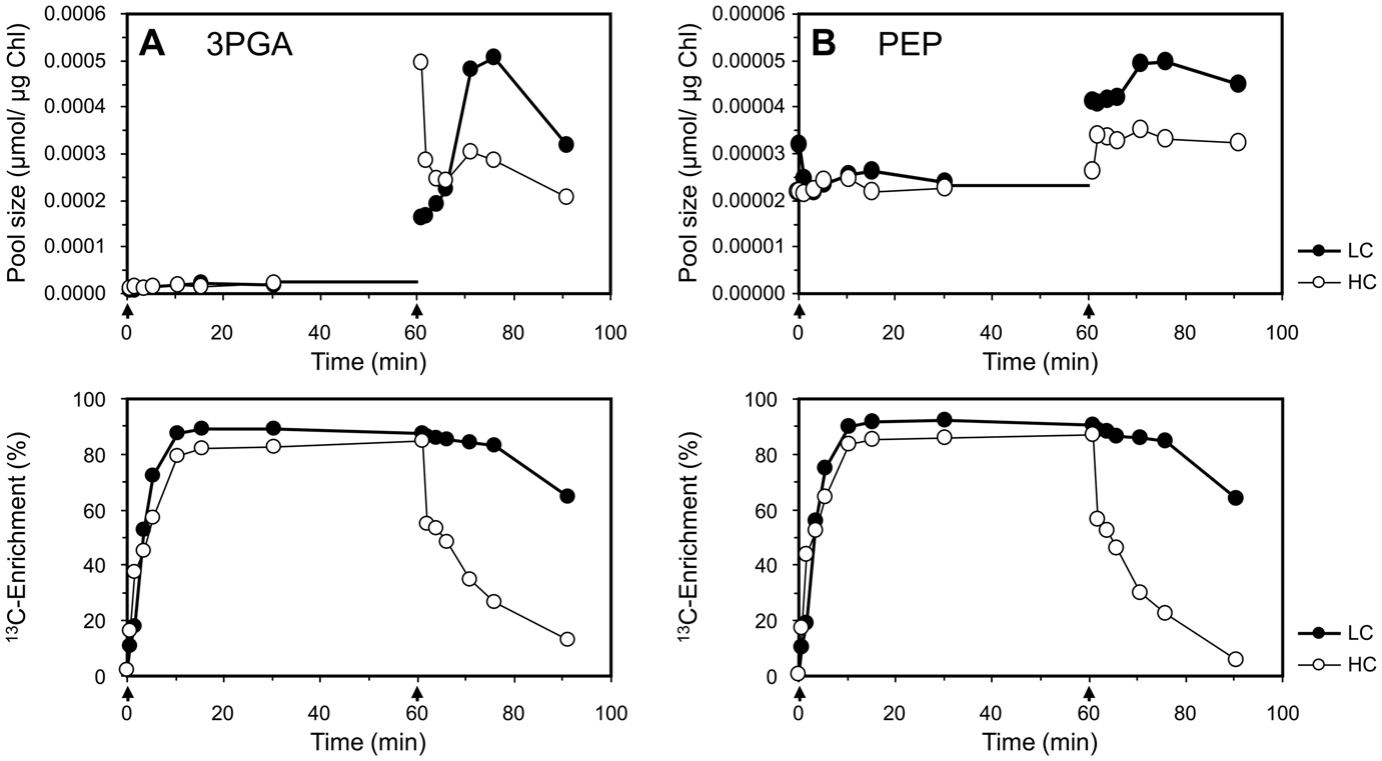
Experimental design of the dynamic metabolic flux analyses in photoautotrophic cultures of *Synechocystis*. The cells were monitored by combined pool size and ^13^C enrichment analyses of (A) 3-phosphoglycerate (3PGA) and (B) phosphoenolpyruvate (PEP). The wild-type strain *Synechocystis* sp. PCC 6803 was probed after pre-acclimation to HC or LC conditions using a very high (2% w/w) ^13^C-NaHCO_3_ (VHC) pulse and a chase following media exchange and aeration with 5% CO_2_ (HC) or 0.035% CO_2_ (LC) of ambient isotope composition (cf. supplementary Methods S1). HC and LC chase conditions were the same as the respective pre-acclimation conditions. Arrows indicate initial sampling at ∼0.5 min after label exchange for pulse or chase. The pool size behaviour (top) and ^13^C enrichment data (bottom) are presented.

Pulse labelling of photoautotrophic *Synechocystis* cultures was performed by adding aliquots of a saturated solution of ^13^C labelled NaHCO_3_ to a final concentration of 2% (w/w). This procedure resulted in a very high carbon (VHC) pulse and was chosen to ensure a step change with the highest ^13^C enrichment possible. Moreover, the VHC conditions should suppress the oxygenase activity of Rubisco. We combined the ^13^C_i_ pulse of stably labelled bicarbonate with a chase using unlabelled CO_2_. Our experimental procedure generated an optimal rectangular step change during the ^13^C_i_ pulse and sufficient enrichment for short isotope dilution times of 10–60 min (Figure 1). In the following study, we focused on the metabolite pools that reached high ^13^C enrichment and thus allowed optimal GC-MS-based analysis. The VHC pulse was applied to cells that pre-acclimated to 5% CO_2_ (HC) or to 0.035% CO_2_ (LC) conditions, as previously reported [15]. For the chase, a quick medium exchange was performed. The cells were subsequently incubated under continuous aeration with either HC or LC identical to the initial pre-acclimation. Our previous work on metabolic profiling revealed highly reproducible metabolic patterns after HC or LC acclimation and indicated that near-steady-state conditions could be achieved using standardised batch cultivation [15].

To analyse changes in carbon partitioning within the primary metabolism, we combined metabolic flux and pool size assessments as previously suggested [17]. The metabolomic data were extended by parameters derived from mass isotopomer distribution analyses, i.e., the initial rate of ^13^C accumulation during the first 10 min and the maximum ^13^C enrichment at 20–60 min. Both parameters were calculated from the transient labelling kinetics of each of the monitored metabolite pools. Based on the analysis of 3PGA as the main entry point of the ^13^C label in photoautotrophic cultures of *Synechocystis*, we estimated the general features of the chosen experimental design. The 3PGA levels did not change after pre-acclimation to HC and LC or throughout the VHC pulse but increased clearly upon initiation of both the HC and LC chase (Figure 1A). As both chase conditions represented a shift to lower C_i_ availability, these observations were in agreement with the previously observed increase of the 3PGA pool 3 h after a shift from HC to LC [15]. In parallel, the transient ^13^C labelling of the 3PGA pool was assessed during pulse and chase. Under our labelling conditions, the 3PGA pool reached its maximum saturated ^13^C enrichment 10 min after the pulse in both the HC and LC acclimated cells. LC cells showed a more rapid ^13^C accumulation. During the chase period with HC conditions, labelling returned below 10% within 30 min. Under LC conditions, a delayed chase response was observed. As expected, the empirical mass distribution vectors of 3PGA and PEP indicated homogenous ^13^C labelling. The kinetic behaviour of the mass distribution vectors (Figure S1) was generally in agreement with the modelled predictions reported earlier [19].

The presence of unlabelled ambient CO_2_ resulted in a perceptible isotope dilution compared to the 98% enrichment of the applied NaHCO_3_. For example, the final ^13^C enrichment in the 3PGA pool was on average >82% and >89% for the HC and LC conditions, respectively (Table S1). Moreover, the label exchange for the chase phase indicated a step change only for the HC condition. Under LC conditions, the label exchange was clearly delayed (Figure 1). Dilution and carryover effects at chase initiation were unavoidable in our hands, especially when implementing the LC chase conditions. Therefore, the slow return of ^13^C label in 3PGA could be caused by both an insufficient physical dilution of ^13^C label and by a physiological effect of the CCM, which is activated under LC conditions but suppressed under HC conditions [12]. To account for these effects, we used the enrichment data of the first assimilation products, for example 3PGA, to correct the influence of ambient CO_2_ and any potential label carryover.

In this study, we analysed the pool size and labelling kinetics as ^13^C enrichment parameters of the observed metabolite pools. Each of these parameters will first be reported in context of the HC versus LC pre-acclimation for the WT strain. Subsequently, the respective phenotypes of the mutant strains will be presented. We focused on the mutants *ΔglcD1* (*Δsll*0404), which is deficient for one of the two isoforms of glycolate dehydrogenase, and *ΔgcvT* (*Δsll*0171), deficient for the T-protein subunit of the glycine decarboxylase complex.

### The entry points of the ^13^CO_2_ label: 3PGA and PEP

**Figure 2:**
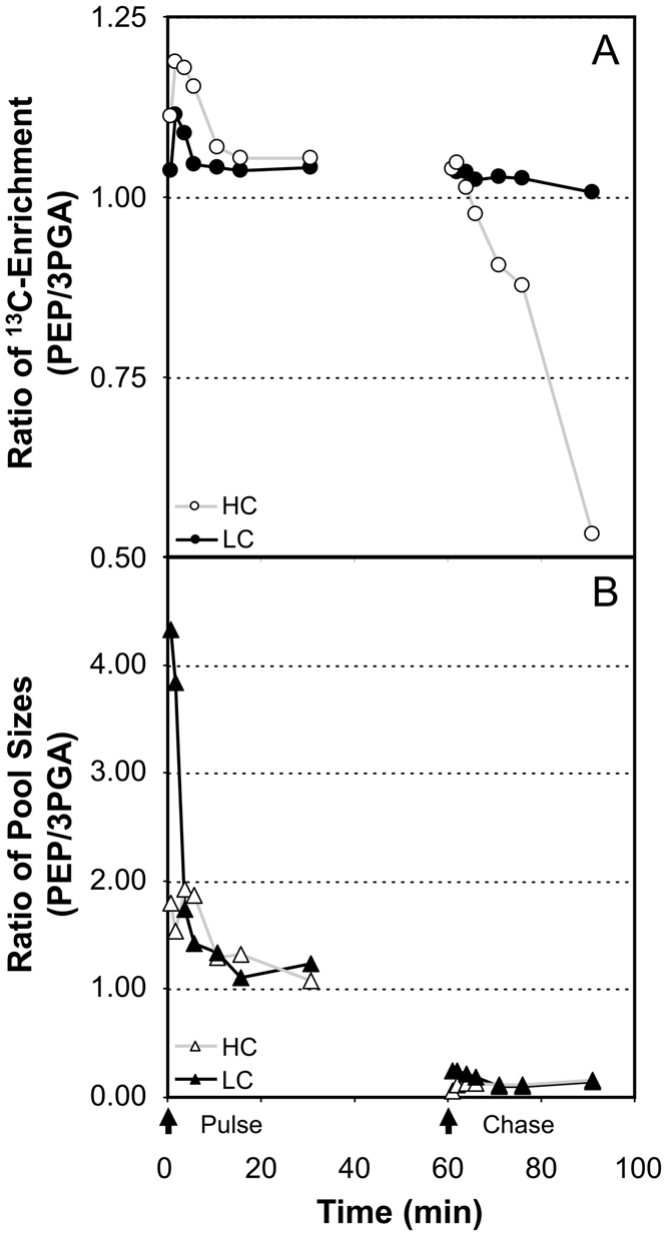
Ratios of PEP/3PGA ^13^C enrichment (A) and respective pool sizes (B). The pulse and chase conditions used in HC and LC acclimated *Synechocystis* sp. PCC 6803 wild-type were identical to the conditions reported in the legend of Figure 1. The initiation of the pulse (0 min) and chase (60 min) are indicated by arrows.

The two most rapidly and completely labelled metabolite pools were the 3PGA pool, as expected, and, unexpectedly, the PEP pool (Figure 1). The ^13^C enrichment was higher and the rate of ^13^C labelling was faster in the PEP pool compared to the 3PGA pool under all conditions and in all analysed mutants. The Student's t-test of the differences in ^13^C enrichment at 20–60 min after the pulse and in the initial ^13^C accumulation rate at 0.5–10.0 min showed that these differences were highly significant in both cases, p = 0.00008 and p = 0.050, respectively. These results are documented in more detail by the PEP/3PGA ratios of both measurements, which were consistently >1.0 (Table 1). The kinetic analysis of the PEP/3PGA ratio of ^13^C enrichment (Figure 2) demonstrated that the PEP pool was labelled faster in the pulse phase and was also de-labelled faster in the chase phases. This observation coincided with a general increase in both pool sizes upon shift from VHC during the pulse phase to HC or LC during the chase phase (Figure 1). The 3PGA pool increased by more than the PEP pool, as demonstrated by a reduction of the PEP/3PGA pool size ratio from above 1 to about 0.15 (Figure 2).

**Table 1:** Rate of ^13^C accumulation during a 0.5–10.0 min ^13^C_i_-VHC pulse using 2% (w/w) ^13^C-NaHCO_3_ and ^13^C enrichment at maximum labelling (20–60 min) in the pools of 3-phosphoglycerate (3PGA) and phosphoenolpyruvate (PEP).

Genotype	Condition	Rate of ^13^C Accumulation		^13^C Enrichment	
		atom% min^−1^		atom%	
		Avg	RSD (%)	Avg	RSD (%)
**3PGA**					
WT	HC	11.1	49.2	86.1	7.2
	LC	15.1	15.1	91.9	4.2
*ΔglcD1* (*sll*0404)	HC	8.6	23.0	74.2	6.5
	LC	13.9	6.6	85.3	6.9
*ΔgcvT* (*sll*0171)	HC	9.8	2.9	84.1	7.3
	LC	14.9	0.9	90.0	0.4
**PEP**					
WT	HC	12.8	38.7	92.3	5.1
	LC	16.7	17.1	95.6	4.0
*ΔglcD1* (*sll*0404)	HC	10.8	26.2	83.1	9.5
	LC	16.1	8.8	92.2	9.2
*ΔgcvT* (*sll*0171)	HC	12.6	10.1	92.2	12.0
	LC	18.3	7.0	95.7	3.9
**PEP/3PGA ratio**		**Rate of ^13^C Accumulation**		**^13^C Enrichment**	
		fold	RSD (%)	fold	RSD (%)
WT	HC	1.20	13.37	1.07	3.41
	LC	1.11	5.95	1.07	3.08
*ΔglcD1* (*sll*0404)	HC	1.25	3.27	1.14	3.84
	LC	1.17	15.38	1.10	4.00
*ΔgcvT* (*sll*0171)	HC	1.28	7.23	1.09	2.74
	LC	1.23	6.01	1.06	2.62

The PEP/3PGA ratios were calculated from paired observations within each sample. Cultures were pre-acclimated and subjected to a chase of either 5% CO_2_ (HC) or 0.035% CO_2_ (LC) of ambient isotope composition. Note that compared to the HC condition, the LC condition resulted in faster and higher labelling of both the 3PGA and PEP pools. Also note that the PEP pool exhibited more rapid and higher labelling than the 3PGA pool. Each replicate experiment (WT n = 3, mutants n = 2) was an independent time series of 7–8 time points sampled ∼0.5–60.0 min after the pulse.

Cells pre-acclimated to the HC or LC conditions exhibited several characteristic differences in 3PGA and PEP labelling, but the initial rate of ^13^C accumulation and the maximum ^13^C enrichment of both metabolites remained strictly correlated during the pulse phase. Firstly, under LC conditions, the initial rates of ^13^C accumulation in both metabolite pools were faster by a factor of ∼1.3 as compared to HC conditions. Also the final ^13^C enrichment was ∼1.1-fold higher (cf. Table 1) with LC conditions. Secondly, the chase phase revealed a substantial difference. While metabolites in cells from both conditions behaved according to the assumption of a rectangular step change after the pulse, only metabolites under the HC chase conditions appeared to follow this assumption after the chase. In contrast, the LC cultures retained high ^13^C enrichment during the first 20 min of the chase until the onset of a delayed and slow de-labelling. This irregular behaviour probably resulted either from the physical carryover effect of ^13^C label from the preceding VHC pulse, as reported above, or from the intracellular CCM activity of *Synechocystis*, which is highly active under LC conditions [12] and should favour the re-assimilation of the ^13^CO_2_ generated from internal sources.

The 3PGA and PEP labelling of the glycine decarboxylase-deficient *ΔgcvT* mutant strain was similar to that of the WT strain. In contrast, the glycolate dehydrogenase mutant, *ΔglcD1*, showed large differences in 3PGA labelling but only slight changes in PEP ^13^C enrichment kinetics. Specifically, the rate of ^13^C accumulation and the final ^13^C enrichment in the 3PGA pool were lowest in the *ΔglcD1* mutant (Table 1). This observation could be explained by the toxic effects of mutant-specific glycolate accumulation on Calvin-Benson cycle activities. These findings were in agreement with previous observations of reduced photosynthetic and growth rates for glycolate dehydrogenase-deficient mutants [14].

### Monitoring the flux into the glucose-6-phosphate and sucrose pools

To analyse the downstream fate of the initial labelling products, ^13^C partitioning into the glucose, glucose-6-phosphate (G6P) and sucrose pools was monitored. The maximum ^13^C enrichment of the glucose pool was unchanged, i.p. 16% for LC (n = 2) and 13% for HC (n = 2). In contrast, the pools of both G6P and sucrose were rapidly labelled during the pulse and de-labelled during the chase. The maximum ^13^C enrichment of G6P was 82% for LC (n = 3) and 76% for HC (n = 3). The maximum ^13^C enrichment of sucrose was slightly lower at 68% for LC (n = 3) and 65% for HC (n = 3). Sucrose labelling was slightly delayed compared to G6P after the pulse, and sucrose de-labelling was consistently more rapid under the LC chase conditions (Figure S2). The differences between G6P and sucrose were less obvious under the HC chase conditions. Taken together these observations indicated a carbon flux from 3PGA via G6P towards sucrose synthesis when *Synechocystis* was exposed to excess C_i_ during the VHC pulse conditions. Furthermore, sucrose was mobilised when the cells were shifted back to lower carbon conditions. The sucrose flux appeared to represent one of the primary paths of photosynthetic CO_2_ assimilation under our conditions. The sucrose flux results (Table 2) were in agreement with earlier findings [14], that the acclimation of *Synechocystis* to the LC condition leads to a higher rate of photosynthesis and sucrose flux than acclimation to HC.

**Table 2:** Sucrose flux estimated from the initial kinetics of a 0–5 min ^13^C_i_-VHC pulse.

Genotype	Condition	Sucrose Flux	
		µmol/min·µg chlorophyll a	
		Avg	RSD (%)
WT	HC	4.72E-05	86.2
	LC	1.38E-04	104.3
*ΔglcD1* (*sll*0404)	HC	1.80E-04	138.4
	LC	2.25E-04	127.6
*ΔgcvT* (*sll*0171)	HC	6.63E-05	141.0
	LC	1.29E-04	138.1

Each replicate experiment (WT n = 3, mutants n = 2) was an independent time series of 7–8 time points sampled ∼0.5–60 min after the pulse. The sucrose flux was calculated based on the influx of carbon from the G6P pool according to previously described methods [39].

Cultures were pre-acclimated to 5% CO_2_ (HC) or 0.035% CO_2_ (LC) of ambient isotope composition.

### Monitoring photorespiratory flux: Glycolate, glycine and serine

In our study, glycolate was the first photorespiratory intermediate in the pathway that was amenable to ^13^C tracing analysis. The 2PG pool was typically at or below detection limits. As was expected of WT cells, the initial rate of ^13^C accumulation and the maximum ^13^C enrichment of the glycolate pool were low under VHC pulse conditions. Nevertheless, even under these conditions, we formally demonstrated ^13^C labelling of the glycolate pool with ∼0.6 atom% min^−1^ for HC conditions and up to ∼1.4 atom% min^−1^ for LC conditions. The glycolate/3PGA ratios of these measurements were ∼0.09-fold and ∼0.18-fold, respectively (Table S1A). Under the HC and LC chase conditions, however, the glycolate pool dropped below the detection limits in WT cells.

**Figure 3:**
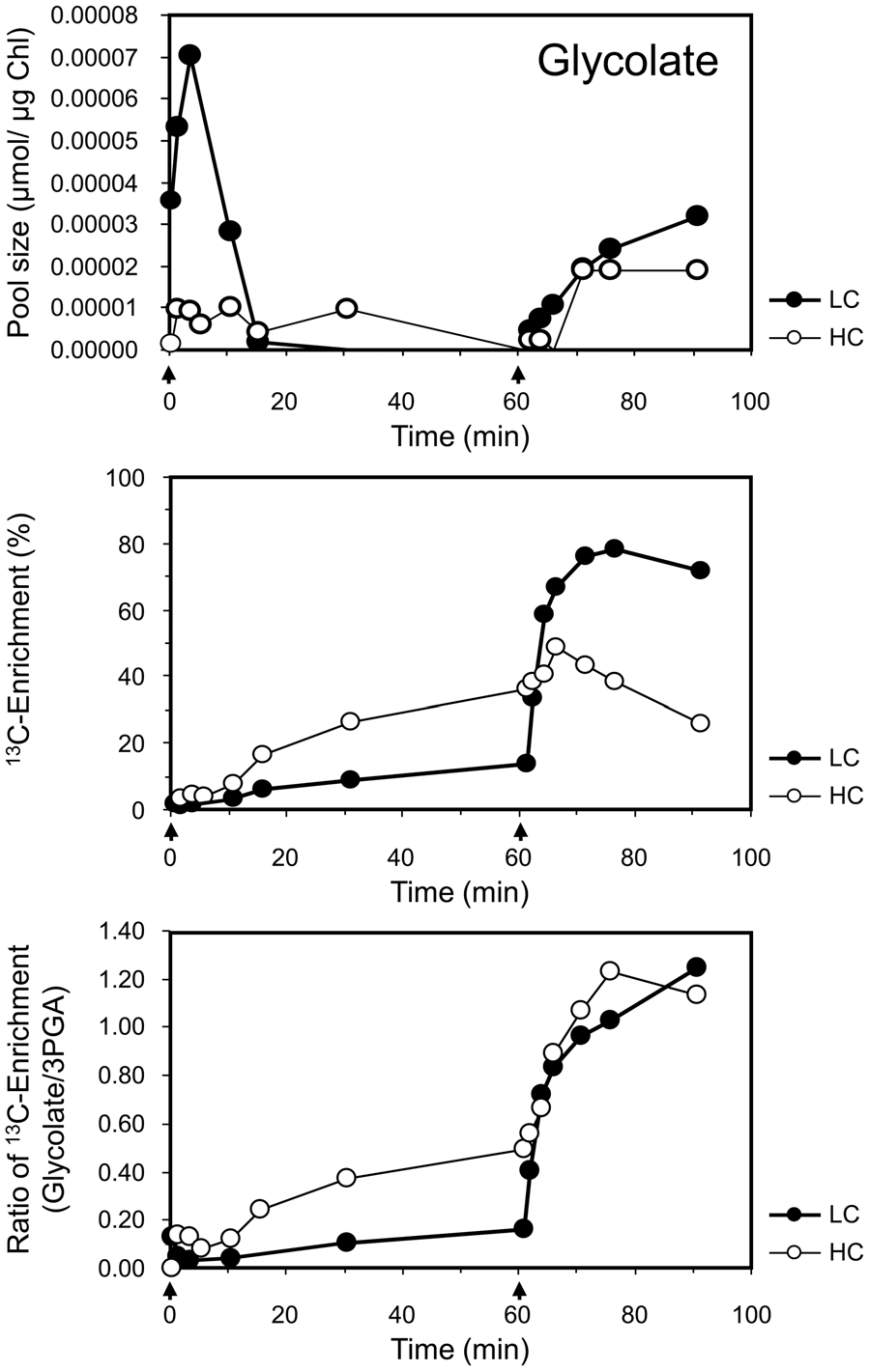
Glycolate pool size, ^13^C enrichment, and ratio of glycolate/3PGA ^13^C enrichment. The data were acquired during the pulse and chase phases under HC and LC conditions with the glycolate-accumulating *ΔglcD1* (*Δsll*0404) mutant. Note the strong increase in the glycolate/3PGA ^13^C enrichment ratio under both the HC and LC conditions (cf. Table S1). The initiation of the pulse (0 min) and chase (60 min) are indicated by arrows.

The *ΔglcD1* mutant had previously been shown to exhibit a large increase in the glycolate pool, as shown by Eisenhut et al. [15], and therefore allowed detailed analysis of glycolate labelling. The glycolate pool size in the *ΔglcD1* mutant responded to the reduced CO_2_ availability under HC and LC chase conditions (Figure 3). Initially, ^13^C rapidly partitioned into the constantly increasing glycolate pool. To compare the HC and LC chase responses, the kinetics of the glycolate/3PGA ^13^C enrichment ratio were analysed in the *ΔglcD1* mutant. If glycolate were predominantly or exclusively generated by the oxygenase activity of Rubisco, we would expect nearly identical glycolate/3PGA ratios under both HC and LC conditions, even though the apparent chase kinetics of 3PGA ^13^C enrichment were different. This expectation is shown to be true in Figure 3 (bottom).

Furthermore, we attempted to quantify the flux of carbon into the glycolate and 3PGA pools under LC and HC chase conditions in the *ΔglcD1* mutant. The glycolate turnover time was shorter than the 3PGA turnover time under LC conditions; specifically, it was 2.9 min (glycolate; k = 0.3337 min^−1^) compared to 90.9 min (3PGA; k = 0.0062 min^−1^). In contrast, the turnover times were in the same order of magnitude under HC conditions, i.e., 39.8 min (glycolate; k = 0.0251 min^−1^) and 25.1 min (3PGA; k = 0.0399 min^−1^). Taking the pool sizes of both metabolites into consideration, we calculated the molar rates of appearance under chase conditions, which may serve as estimates of the rates of oxygenation (glycolate) and carboxylation (3PGA). Under the LC condition, the molar rates of appearance were 4.80 * 10^−6^ µmol min^−1^ µg^−1^ Chl and 1.21 * 10^−6^ µmol min^−1^ µg^−1^ Chl for glycolate and 3PGA, respectively. The ratio of the molar rates of appearance was ∼4.0 (glycolate/3PGA). *Synechocystis* cells in the HC condition showed a 100-fold lower ratio (∼0.04 glycolate/3PGA). The molar rates of appearance were 4.27 * 10^−7^ µmol min^−1^ µg^−1^ Chl and 1.18 * 10^−5^ µmol min^−1^ µg^−1^ Chl for glycolate and 3PGA, respectively.

**Figure 4:**
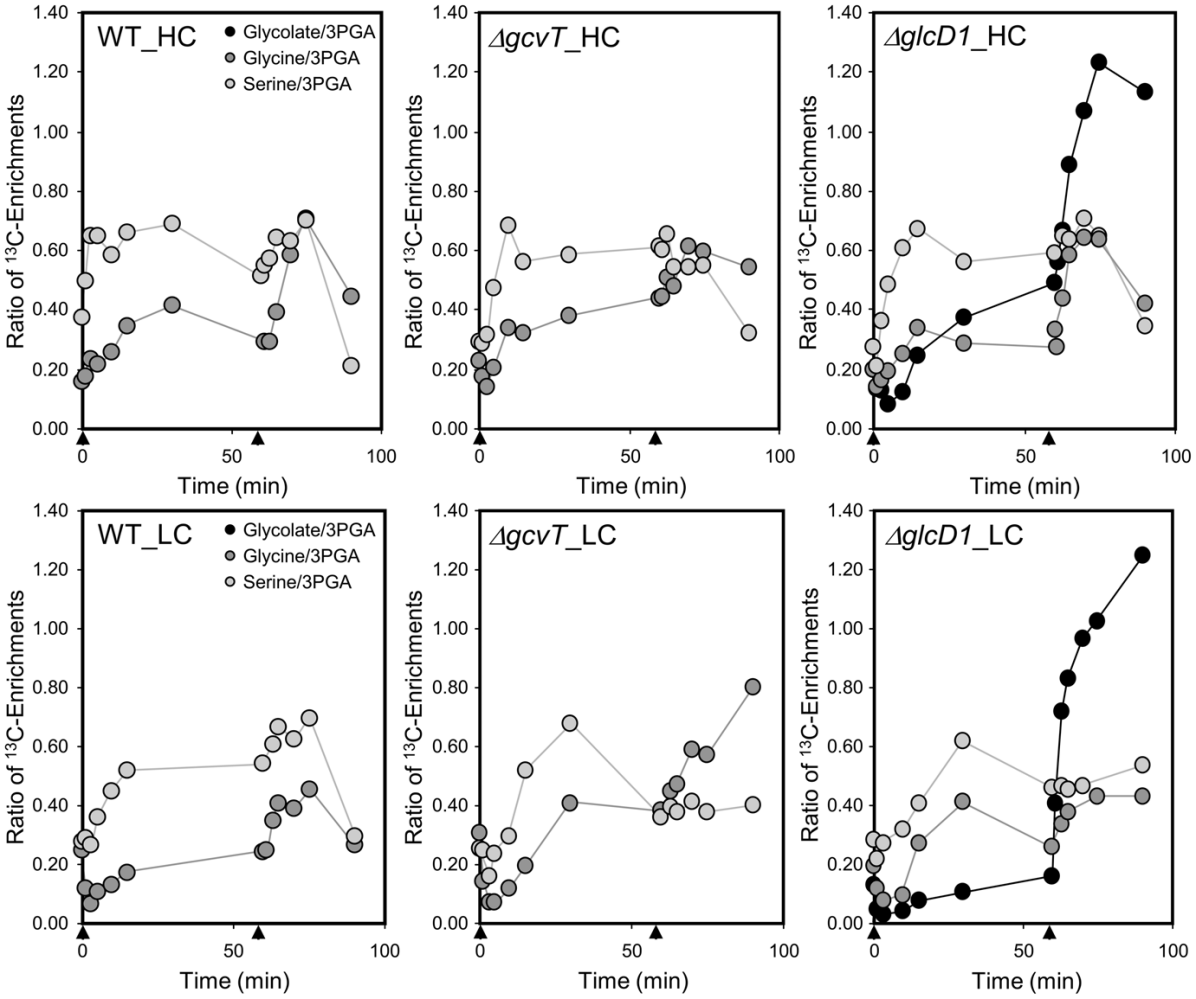
Ratios of glycine and serine ^13^C enrichment to 3PGA ^13^C enrichment. Pulse and chase phases under HC conditions (top) and LC conditions (bottom) were used with the wild-type (left), the *ΔgcvT* (*Δsll*0171) mutant (middle) and the *ΔglcD1* (*Δsll*0404) mutant (right) strains. Glycolate data from the *ΔglcD1* mutant are included in the right panel. The initiation of the pulse (0 min) and chase (60 min) are indicated by arrows.

Moreover, the photorespiratory pathway was monitored with respect to the ^13^C enrichment in the glycine, serine, and glycerate pools. Again, ratios of ^13^C enrichment to 3PGA were used because this approach corrects for the differences in 3PGA labelling among the different experiments (Figure 4). The serine ^13^C enrichment ratios of WT cells were higher than the glycine ^13^C enrichment ratios throughout the VHC pulse (Figure 4, left panel). The ^13^C enrichment ratios of glycerate were equal to or less than the ^13^C enrichment ratios of serine (data not shown). These observations were consistent under all conditions and in all genotypes (Figure 4). Our observations thus indicated that the synthesis of serine from glycine was minimal under VHC conditions, while the conversion of glycerate to serine or the opposite reaction seemed to occur under HC and LC conditions.

During the chase periods, the glycolate-accumulating *ΔglcD1* mutant showed a transient increase in glycine and serine ^13^C enrichment ratios that was similar to the effect observed in the WT (Figure 4, right panel). The *ΔgcvT* mutant, which is deficient in glycine decarboxylase and is known to accumulate glycine under LC conditions [15], showed the expected enhanced accumulation of the ^13^C label in the glycine pool under LC chase conditions (Figure 4, central panel).

### Monitoring PEP utilization: Aspartate, malate and citrate

**Figure 5:**
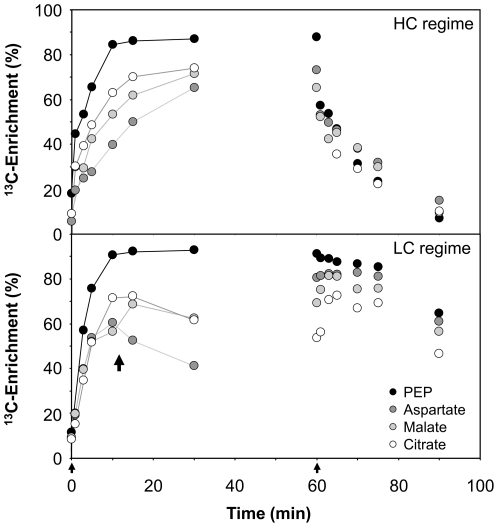
Aspartate, malate, and citrate ^13^C enrichments compared to phospoenolpyruvate (PEP) ^13^C enrichment. Data were obtained under the HC and a LC conditions with *Synechocystis* sp. PCC 6803 wild-type cultures. During the ^13^C_i_-VHC pulse with LC acclimated cells, a transient mobilisation of an internal carbon source of ambient mass isotopomer composition was apparent (marked by the arrow within the lower panel). Carbon of ambient mass isotopomer composition appears to enter the aspartate pool first and to persist longest within the citrate pool. The initiation of the pulse (0 min) and chase (60 min) are indicated by arrows.

Oxaloacetate, the product of PEP carboxylase activity, was not measured in our study. Instead PEP utilisation was monitored by assessing the kinetics of ^13^C enrichment in three metabolic oxaloacetate products: aspartate, malate, and citrate (Figure 5). To normalise the ^13^C labelling of these pools, we calculated ratios of the initial rate of ^13^C accumulation and maximum ^13^C enrichment with respect to PEP instead of the previously chosen 3PGA (Table S1B).

During the VHC pulse, ^13^C accumulation was highest in the citrate pool, followed by the malate and aspartate pools. Unexpectedly, the order changed over the monitored time (Figure 5). At the onset of the pulse phase, ^13^C enrichment was highest in the aspartate pool, followed by the malate and citrate pools. The reversal of ^13^C enrichment was noticeable in cells pre-acclimated to HC and became obvious under LC conditions. The LC condition also demonstrated the mobilisation of unlabelled carbon during the early phase of the VHC pulse. This internal carbon source first entered the aspartate pool (Figure 5, arrow) and appeared to persist for the longest time in the citrate pool. Under the same conditions, the ornithine analyte showed essentially the same internal carbon mobilisation effect as aspartate but with lower ^13^C enrichment (data not shown). Note that due to chemical conversions during the derivatisation of metabolites for GC-MS analysis, the measured ornithine analyte represents the sum of the ornithine, arginine, and citrulline pools.

Indications of additional ^13^C fixation through PEP carboxylase activity were found by analysing the ratios of the initial rates of ^13^C accumulation for the various compounds with respect to PEP (Table S1B). In WT cells, the 3PGA/PEP, citrate/PEP, and malate/PEP ratios of the initial rates of ^13^C accumulation were ∼0.96, ∼0.78, and ∼0.62, respectively. These ratios were not influenced by pre-acclimation of the WT cells to the HC or LC conditions. The aspartate/PEP ratio was, however, increased by 1.34 fold under the LC condition (0.65) compared to the HC condition (0.48). This LC acclimation effect was even more apparent in the two photorespiratory mutants, *ΔglcD1* and *ΔgcvT*, with aspartate/PEP ratios approximating 1 under LC conditions in both mutants. A similar but less extreme effect in the mutants was observed for the malate/PEP ratios. These ratios increased by ∼1.3-fold in cells pre-acclimated to LC compared to those receiving HC. The citrate/PEP ratios showed the same tendency under the LC condition and were only marginally increased in the mutant strains compared to WT. Under HC, the citrate/PEP ratios were, however, reduced in both mutants compared to WT.

## Discussion

### The major paths of carbon assimilation in *Synechocystis*

**Figure 6:**
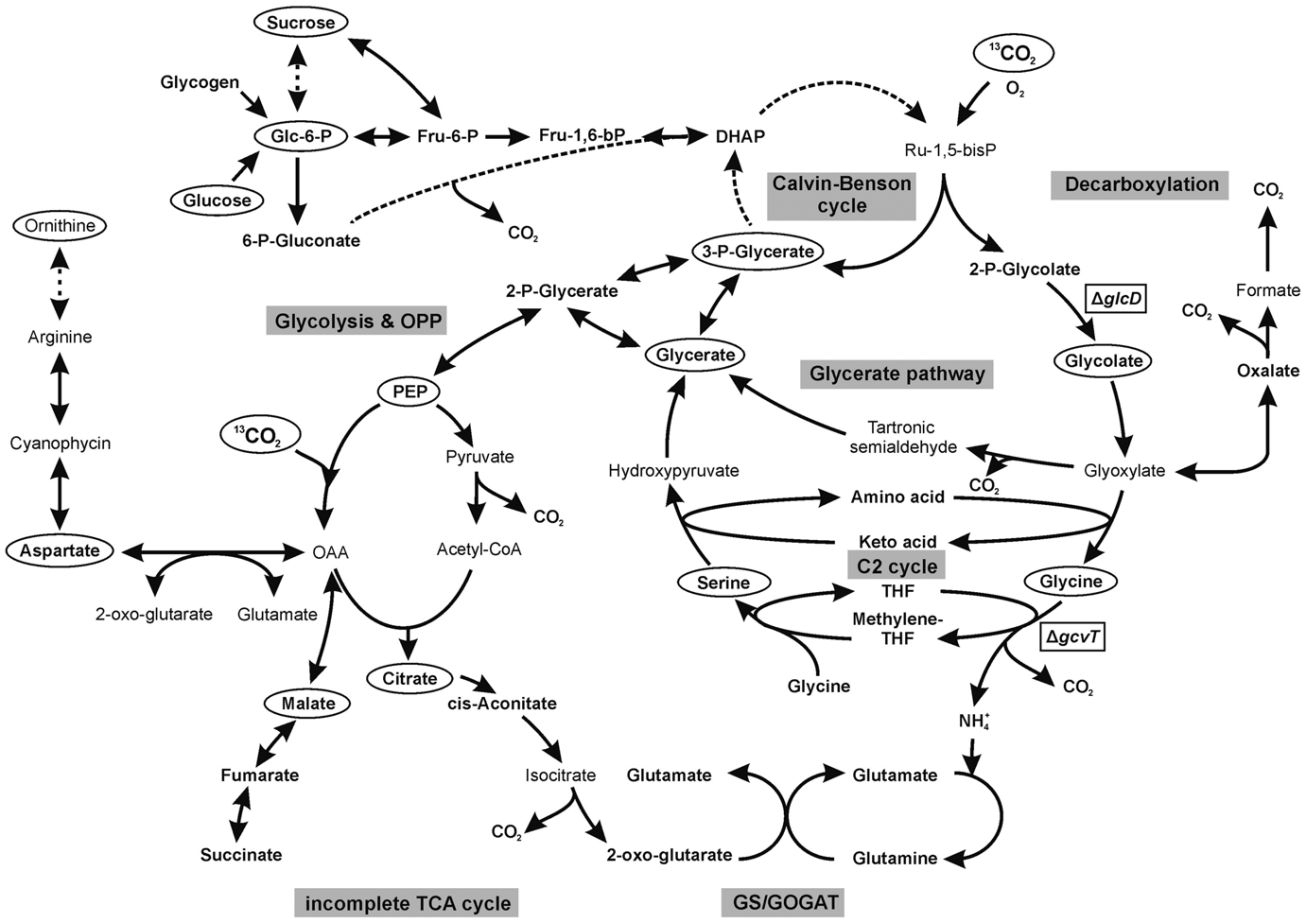
Scheme of central carbon and nitrogen metabolism in *Synechocystis* sp. strain PCC 6803. Ellipses highlight the ^13^C labelled metabolites detected and evaluated in this study. The enzymatic steps affected in the *ΔglcD* and *ΔgcvT* mutants are marked by boxes.

Our data were essentially consistent with the established fact that 3PGA is the first assimilation product of the Calvin-Benson cycle. Rapid labelling of 3PGA with ^13^C suggests that C3-photosynthesis is the dominant carbon fixation pathway in *Synechocystis*. Fixation of ^13^C_i_ via PEP carboxylase seems to be less important, but occured in LC acclimated cells (see below). Following the dilution of the initial ^13^C label of 3PGA into the metabolite pools that were measured by our GC-MS-based technology, we observed two major branches of carbon flow (Figure 6). The first branch leads from 3PGA to sucrose via G6P with minimal leakage of carbon into the free glucose pool. The flux of carbon from 3PGA through this branch into sucrose appears to reflect the photosynthesis rate of the respective pre-acclimated cells [15] because the enrichment of the sucrose pool is higher in LC cells than in HC cells (cf. Table 2). The observed conversion of 3-PGA into sucrose and probably also into glycogen shows that surplus carbon is converted into storage compounds. The second major path of carbon appears to lead to aspartate, malate, and citrate (Figure 6) via PEP and probably further into the amino acid pools via 2-oxo-glutarate. This second branch of 3PGA utilisation directly fuels the biosynthesis of primary metabolites, specifically amino acids that are generated via the incomplete tricarboxylic acid cycle of *Synechocystis*. In agreement with the interruption of the canonical tricarboxylic acid cycle in *Synechocystis*, fumarate and succinate were labelled more slowly than malate (data not shown).

We further conclude that C4 carboxylation via PEP carboxylase also occurs under our experimental conditions, specifically in LC acclimated cells. The joint action of C3 and C4 carboxylation via Rubisco and PEP carboxylase, respectively, has been revealed by ^14^C labelling experiments using different cyanobacterial strains, as shown by Döhler [20]. However, the relative ratio is still a matter of discussion. The oxaloacetate product, which cannot directly be measured by our current technique, appears to be rapidly converted into aspartate and malate via the aspartate aminotransferase and malate dehydrogenase reactions. Of the potential oxaloacetate products, citrate is the most rapidly labelled, possibly because it receives part of its carbon backbone via acetyl-CoA. Acetyl-CoA should be highly labelled because it is generated from the rapidly labelled PEP pool via the final steps of the glycolytic pathway. However, as judged by the rates of ^13^C accumulation, the C3 carboxylating pathway from 3PGA via PEP appears to be the main route to transfer newly fixed organic carbon into the central metabolism of *Synechocystis*. The ^13^C enrichment of oxaloacetate products shows that primary ^13^C_i_ fixation via Rubisco is dominant under our experimental conditions.

One of the unexpected results was the observation that first aspartate and then malate and citrate receive ^12^C from internal stores during the ^13^C_i_-VHC pulse (Figure 5). We interpret this observation as a carbon mobilisation from internal sources into the aspartate and ornithine/arginine/citrulline pools. The source could be fast cyanophycin degradation. Cyanophycin is a storage polymer in cyanobacteria that has a poly-aspartate backbone and arginine side chains. Cyanophycin accumulates mainly as a response to excess nitrogen in *Synechocystis* [21]. The VHC pulse results in an environment characterised by high C_i_ availability and relatively low nitrogen. This should apply especially to LC cells, which were pre-acclimated to rather high C/N conditions. Cyanophycin degradation mobilises stored nitrogen and may ultimately lead to rebalancing of the intercellular C/N ratio. Such a mobilisation process should be considered in future studies that aim at dissecting the relative contribution of C3 and C4 metabolism to the biosynthesis of organic acid intermediates such as citrate.

### Indications for carbon channelling from 3PGA into the PEP pool

Had Calvin and Benson [22], [23] used *Synechocystis* to unravel the photosynthetic carbon cycle, PEP would have likely been misinterpreted as the first assimilation product of the carboxylation reactions. We found clear evidence that the PEP pool of *Synechocystis* is not only more rapidly labelled but also has a slightly but significantly higher final ^13^C enrichment than the 3PGA pool under all of our experimental conditions (Table 1, Figure 2). Even today, this phenomenon could be interpreted as evidence for the presence of alternative CO_2_ fixation mechanisms in cyanobacteria. Such pathways for CO_2_ fixation, e.g., the reductive tricarboxylic acid cycle or the carbamoylphosphate synthetase pathway, have previously been suggested to be operative in cyanobacteria [24]. Both paths have been implicated for ancillary CO_2_ fixation. In view of the incomplete tricarboxylic acid cycle in *Synechocystis* and considering our data, i.e., the lower rates of ^13^C accumulation in the citrate and malate pools compared to the PEP pool, we consider it unlikely that under our growth conditions *Synechocystis* utilizes any of the above ancillary routes of CO_2_ fixation or the hydroxypropionate pathway for CO_2_ fixation, which was recently discovered in *Chloroflexus aurantiacus* [25]. This view is supported by the absence of genes for such alternative C fixation pathways from the complete genome sequence of *Synechocystis* (CyanoBase at http://genome.kazusa.or.jp/cyanobase/Synechocystis).

Instead, we favour the interpretation that the majority of newly assimilated carbon is channelled from 3PGA to PEP, and the much smaller PEP pool is more quickly saturated with ^13^C. With this interpretation, we need to assume that two sub-pools of 3PGA exist in *Synechocystis*. The carboxysome, a cyanobacterial micro-compartment that is a component of the CCM and harbours most of the cellular Rubisco, may well be a rapidly labelled 3PGA pool from which PEP is produced. The second 3PGA pool may be cytosolic. The cytosolic 3PGA pool could originate from a second pool of Rubisco. Rubisco was recently indicated to exist outside of the carboxysome but attached to thylakoids and in close association with other Calvin-Benson cycle enzymes [26]. Carbon should be channelled into PEP either within the carboxysomes or along with the export of 3PGA from the carboxysome into the cytosol. The cytosolic 3PGA pool may well be diluted with unlabelled carbon as a result of the turnover or mobilisation of previously assimilated soluble sugars or glycogen. Channelling into PEP may also indicate that the PEP carboxylase reaction for *Synechocystis* is more important than previously described [27]. This conclusion is well supported by the rapid accumulation of assimilated carbon in pools downstream of oxaloacetate (cf. discussion above) and may explain enigmatic early reports of high accumulations of label in unexpected metabolite pools, such as the aspartate pool [20], [24].

### Formal demonstration of photorespiratory flux

We detected a significant partitioning of the ^13^C label into the glycolate pool, primarily in the *ΔglcD1* mutant, but also in WT cells under our VHC pulse conditions. Thus we show that even when *Synechocystis* cells are exposed to very high C_i_ concentrations, the oxygenation reaction of Rubisco is not completely suppressed. This finding is in agreement with the reduced growth and photosynthesis caused by glycolate accumulation in cells of the *ΔglcD1* mutant grown under HC conditions [13].

The glycolate-accumulating *ΔglcD1* mutant and the glycine-accumulating *ΔgcvT* mutant proved to be valuable tools to generate a detailed demonstration of photorepiratory flux (Figure 6). The *ΔglcD1* mutant allowed the demonstration of enhanced carbon flux into the glycolate pool after a shift to lowered C_i_ during the chase phase (Figure 3). In addition, this mutant enabled an estimation of the rates of oxygenation and carboxylation under HC and LC chase conditions by quantification of the molar rates of ^13^C appearance in the glycolate pool compared to the 3PGA pool. An enhanced photorespiratory flux was found under LC conditions compared to HC conditions. In contrast, WT cells displayed a very low photorespiratory flux under ambient CO_2_ conditions, which was consistent with previous reports on the quantitatively low importance of photorespiratory 2PG metabolism [11].

The *ΔgcvT* mutant demonstrated enhanced ^13^C flux into the glycine pool under chase conditions. This evidence may explain the coinciding increase in glycine levels (Figure 4), which is possibly linked to the increased production of glycine and reduced glycine utilisation of this slow-growing mutant [13].

### Mutants and WT compared under HC and LC conditions

HC and LC acclimated *Synechocystis* cells were labelled with ^13^C to assess the differential acclimation to high and limiting C_i_ availability. Labelling of 3PGA and sucrose confirmed previous observations on the respective photosynthesis rates, i.e., that HC acclimation resulted in lower maximal assimilation rates than LC acclimation, and that *ΔglcD* mutants appear to be partially deficient in photosynthesis. Moreover, HC acclimation was linked to greater channelling of assimilate into the PEP pool (Figure 2). The apparent differences between the LC and HC acclimations were most clearly assessed when comparing relative ^13^C enrichment after normalisation to either 3PGA or PEP in the different experiments (Figures 4 and 6). The ratios demonstrated only minor differences of ^13^C partitioning into the glycolate pool after a HC or LC chase in the *ΔglcD1* mutant. In addition, flux through PEP carboxylase appeared to be slightly affected by the different conditions in WT cells. Only an increased aspartate/PEP-labelling ratio was observed in LC acclimated WT cells (Table S1B), which indicates PEP carboxylase activation under LC conditions. In contrast, in addition to the aspartate/PEP ratio, the mutants exhibited increased malate/PEP and citrate/PEP ratios for the initial rates of ^13^C accumulation, which is consistent with an enhanced flux through the PEP carboxylation reaction under LC conditions. The enhanced ^13^C_i_ fixation through PEP carboxylase in both photorespiratory mutants, and to a lesser extent in WT cells, could indicate that this alternative carboxylating enzyme is activated in LC cells to compensate for the inhibitory effects of glycolate or glycine on Rubisco and other Calvin-Benson cycle enzymes.

## Materials and Methods

### Strains and culture conditions

The strain *Synechocystis* sp. PCC 6803 was obtained from Prof. Murata (National Institute for Basic Biology, Okazaki, Japan) and served as the WT for this study. The generation and characterisation of the mutants in the *Synechocystis* sp. PCC 6803 photorespiratory pathway, i.e., *ΔglcD1*, which bears a defect in the glycolate dehydrogenase-coding gene *sll*0404, and *ΔgcvT*, which bears a defect in the gene *sll*0171 that encodes the T-protein subunit of the glycine decarboxylase complex, have been described elsewhere [13], [28]. Cultivation was performed in BG11 medium [29] at pH 8.0 with an initial optical density (OD_750_) of ∼0.8, which is equal to ∼10^8^ cells mL^−1^. Mutants were grown in the presence of 50 μg mL^−1^ kanamycin (Km) or 20 μg mL^−1^ spectinomycin (Sp). Potential contamination by heterotrophic bacteria was ruled out by spreading 0.2 mL of culture on LB plates.

Axenic cultures were grown photoautotrophically at 29°C in batch cultures using 3-cm glass vessels with 5-mm glass tubes for aeration with 5% CO_2_-enriched air (HC) with a bubbling flow rate set to 5 mL min^−1^. Cultures were continuously illuminated with warm light using an Osram L58 W32/3 at 130 μmol photons s^−1^ m^−2^. Pre-cultures were split into equal parts. One part continued to be grown under HC conditions while the second subculture was grown under C_i_ limiting conditions (LC conditions) by bubbling with ambient air containing 0.035% CO_2_. Acclimation to low C_i_ by pre-cultivation with ambient air was performed 24 h prior to the isotope-labelling experiments. The pH of the growth medium was stable under the chosen HC and LC cultivation conditions, which were essentially as described earlier [15].

### Transient ^13^C_i_ isotope-labelling experiments

Pulse experiments with ^13^C_i_ were performed by transferring 40 mL of HC or LC pre-culture into a new cultivation vessel that was kept without bubbling under otherwise identical conditions. The transient isotope pulse was started under very-HC conditions (VHC), i.e. 2% (w/w) sodium hydrogen carbonate, by adding 10 mL of a ^13^C-bicarbonate stock solution prepared by dissolving 1 g sodium hydrogen carbonate 98 atom% ^13^C (Sigma-Aldrich) in 10 mL BG11. The pH of the ^13^C-bicarbonate stock solution was adjusted to 8.0. Subsequent chase experiments were performed after a 60 min pulse according to the pre-acclimation, either under 5% CO_2_ (HC) or 0.035% CO_2_ (LC) conditions. The BG11 medium was exchanged by centrifugation (3000g, 2 min, 22°C), careful removal of the supernatant and re-suspension in the original volume. The fresh medium was pre-adjusted to the ambient ^12^C/^13^C isotope ratio and CO_2_ concentration by bubbling with ambient air. Care was taken to minimise the carryover of ^13^C_i_ upon medium exchange. To enable fast sampling of the chase kinetics, a wash of the cell pellet had to be avoided. Samples were taken immediately after pulse or chase and at 1, 3, 5, 10, 15, 20 (or 30), and 60 min. Observations of pulse and chase kinetics were repeated with 2–3 independent cultivations.

In our hands a clear step change of ^13^C label was only possible using high bicarbonate concentrations at the cost of a change of osmolarity within the growth medium. The use of low enrichments for pulse and chase experiments, which are possible using the highly sensitive radioactive ^14^C detection methods, are not applicable to the less-sensitive ^13^C detection methods. To control for unavoidable imperfect step changes, we normalised the initial rates of ^13^C accumulation and the ^13^C enrichment in downstream metabolite pools of a pathway to the respective parameters of the first ^13^C assimilation products, i.e., 3PGA and PEP (cf. supplementary Methods S1).

### GC-EI-TOF-MS analysis of metabolite pool sizes and mass isotopomer distributions

The previously described GC-EI-TOF-MS metabolite profiling technology for methoxyaminated and trimethylsilylated methanol/water-soluble metabolites from *Synechocystis* [15], [30] was applied to assess a combination of both the metabolite pool sizes and the respective ^13^C mass isotopomer distributions from ^13^C labelled samples. Culture samples of 2–7 mL, equivalent to about 10^9^ cells mL^−1^, were harvested and separated from the media by fast filtration in the light using a glass vacuum filtration device with controlled temperature and illumination. To minimize the time between sample collection and analysis, no wash was performed. This sampling method, which uses a filter disc to remove secreted metabolites and components of the growth medium, has been previously described [31]. In addition, we performed rapid metabolic inactivation by immediately shock-freezing the cells on the filter disk in liquid N_2_ to obtain the quickest possible samples for tracing studies [22], [23]. Recovery checks by internal standardisation using chemically synthesised stable isotope-labelled reference compounds had to be omitted so as not to interfere with the ^13^C tracing experiments. Instead, metabolite pools were normalised to the chlorophyll a content [14]. The relative changes of normalised metabolite pool sizes were based on the peak intensities. In addition to the conventional metabolite profiling procedure, the sum of all observed mass isotopomers of characteristic fragments was calculated to enable pool size quantification in the presence of shifting mass isotopomer distributions. The metabolites glycolate, glycine, serine, glucose-6-phosphate (G6P), sucrose, glycerate, phosphoenolpyruvate (PEP) and 3-phosphoglycerate (3PGA) were externally calibrated using a dilution series of nine concentration points, ranging from 0.04 ng µL^−1^ to 166.67 ng µL^−1^ of a chemically defined mixture of authenticated reference compounds in equal amounts at 1.0 mg mL^−1^ for each compound [32].

### Data processing and compound identification

GC-TOF-MS chromatograms were processed using TagFinder-Software [33]. Analytes were manually identified using the TargetFinder plug-in of the TagFinder-Software and a reference library of ambient and ^13^C labelled mass spectra and retention indices (RI) from the Golm Metabolome Database (GMD, http://gmd.mpimp-golm.mpg.de/) [34], [35]. A peak intensity matrix containing all available mass isotopomers of characteristic mass fragments that represented the primary metabolites under investigation was generated by TagFinder. This matrix was processed using the *CORRECTOR* software tool (http://www-en.mpimp-golm.mpg.de/03-research/researchGroups/01-dept1/Root_Metabolism/smp/CORRECTOR/index.html). Using this batch processing tool, we calculated the sum of mass isotopomer intensities and the ^13^C enrichments of mass fragments that had been annotated previously [17] using previously described methods [36], [37].

### Calculations and statistical data mining

Data management, data transformation, calculations, and statistical analyses were performed using Microsoft Office Excel 2003 software, the R 2.9.1 statistical programming package, and SigmaPlot 11.0 software (Sysstat Software Inc., San Jose, CA, USA). Calculations of the molar rate of appearance (*R_a_*) for the Rubisco reactions were performed according to Wolfe and Chinkes [38] using the equation R_a_ = k*Q, where *Q* represents the pool size of the respective metabolite. To obtain *k*, the turnover rate, the experimental data were fitted to the equations *E_t_ = E_p_(1-e^−kt^)* or *E_t_ = E_0_e^−kt^*. The turnover rate (k^−1^) of a pool is given in min^−1^. E_t_ represents the isotopic enrichment at time t, E_p_ the plateau enrichment and E_0_ the enrichment at t  = 0. The sucrose flux was calculated based on the influx of the precursor G6P, as described by Roessner-Tunali and co-authors [39].

Error estimates and error minimisation are relevant when judging labelling results. The technical precision when determining the ^13^C enrichment of a metabolite pool is generally below 2% relative standard deviation (RSD) when using the GC-TOF-MS metabolite profiling method [17]. In contrast, the technical error of a metabolite pool size determination using the same method is about one order of magnitude higher, in the range of 5–20% RSD [40]. In addition to the technical error, the biological variability, i.e., the culture-to-culture differences of replicated *Synechocystis* experiments, was considered using the 3PGA measurement as a test case. The precision of 3PGA pool size determinations in replicate WT and mutant cultures had previously been determined as 25.6% RSD (n = 9), similar to the average 22.9% RSD (n = 9) of all metabolites observable by GC-TOF-MS profiling (cf. Table S1 of [15]). The culture-to-culture variation of the parameters was smaller. The maximum ^13^C enrichment, determined as atom% at 20–60 min, of the 3PGA or PEP pools had, on average, 6.3% RSD, while the initial rate of ^13^C accumulation, determined as the atom% min^−1^ by linear regression (r^2^ = 0.85–0.99), in either of the pools exhibited, on average, 17.1% RSD, as assessed by triplicate WT and duplicate mutant experiments (Table 1).

## Supporting Information

Methods S1 **Motivation, detailed description and error assessment of the experimental design chosen for the comparative metabolic flux phenotyping of *Synechocystis* sp. PCC 6803 wild-type and photorespiratory mutants.**
(DOCX)

Figure S1 **Head-to-tail view of ambient (red) and maximally ^13^C labeled (blue) mass spectra from the 3-phosphoglycerate (4TMS) and phosphoenolpyruvate (3TMS) analytes of** ***Synechocystis*** **.** Mass fragments suitable for mass isotopomer distribution analysis are indicated by an asterisk [17]. Inserts indicate the identifiers of the Golm Metabolome Database [35] and the expected retention index (RI) and experimental deviation (*Δ*RI%) within *Synechocystis* extracts. The empirical mass distribution vectors of the M-15^+^ fragments, i.e., C_14_H_36_O_7_PSi_4_ (M0-3  =  459-462) from the 3-phosphoglycerate (4TMS) analyte and C_11_H_26_O_6_PSi_3_ (M0-3  =  369-372) from the phosphoenolpyruvate (3TMS) analyte, are shown on the right, as determined from an experiment performed under the HC condition.
(TIF)

Figure S2 **^13^C enrichment of the glucose, glucose-6-phosphate and sucrose pools in the wild-type strain** ***Synechocystis*** **sp. PCC 6803.** Data from LC acclimation followed by a VHC pulse at t  =  0 min and LC chase at t  =  60 min or 120 min are shown. Note that glucose was detectable only under VHC pulse conditions in 2 of 3 replicate experiments.
(TIF)

Table S1 **Initial rates of ^13^C accumulation.** Data were acquired during a 0.5-10.0 min ^13^C_i_ pulse and ^13^C enrichment at maximum labelling (20-60 min) in (A) the glycolate pool compared to the 3PGA or PEP pools and (B) aspartate, malate, and citrate pools compared to the PEP pool. The ratios of the initial rates of ^13^C accumulation and the ^13^C enrichment at maximum labelling were calculated from paired observations within each sample. Cultures were pre-acclimated and subjected to a chase with ambient isotope composition of either 5% CO_2_ (HC) or 0.035% CO_2_ (LC).
(XLS)
