# Supplementary material for: Modulation of the Major Paths of Carbon in Photorespiratory Mutants of Synechocystis
Source: PLoS One. 2011 Jan 21;6(1):e16278. doi: 10.1371/journal.pone.0016278 (PMC3025020; doi:10.1371/journal.pone.0016278)
Supplement: Methods S1 — Motivation, detailed description and error assessment of the experimental design chosen for the comparative metabolic flux phenotyping of Synechocystis sp. PCC 6803 wild-type and photorespiratory mutants. (DOCX) [file pone.0016278.s001.docx]

**Supplementary Methods**

Experimental design of metabolic flux phenotyping

Metabolic flux analysis in *Synechocystis* has been reported previously under mixotrophic or heterotrophic CO_2_-exclusion conditions using [U-^13^C]-glucose as an entry point for the stable isotope label into the cyanobacterial metabolism [1]. These conditions are unlikely to reveal direct insights into the photoautotrophic metabolism of a cyanobacterium. In this study, pulse labelling was performed by adding aliquots of a saturated solution of ^13^C-labelled NaHCO_3_ to a final concentration of 2% (w/w). This procedure resulted in a very high carbon (VHC) pulse and was chosen to ensure a step change with the highest ^13^C enrichment possible. The pH was kept constant throughout the experiment. Because of feasibility considerations, we chose batch cultivation under continuous illumination. Such a system was previously used to profile changes in primary metabolism [2]. We performed transient stable-isotope labelling experiments by combining a ^13^C-pulse with stably labelled bicarbonate with a chase using CO_2_ of ambient isotope composition. Such an approach has recently been suggested and theoretically assessed [3].

Our experimental procedure generated an optimal rectangular step change during the ^13^Ci-pulse and sufficient enrichment in short isotope dilution times of 10-60 min (cf. Fig. 1). The VHC pulse was applied to cells that were pre-acclimated to either 5% CO_2_ (HC) or 0.035% CO_2_ (LC) conditions as previously reported [2]. For the chase, we performed a media exchange using fast centrifugation followed by continuous aeration with either 5% CO_2_ (HC) or 0.035% CO_2_ (LC) under conditions identical to the initial pre-acclimation. These experimental settings minimised the effects of long-term CO_2_ acclimation responses. Our previous work on metabolic profiling revealed highly reproducible metabolic patterns after HC or LC pre-acclimation and indicated that near steady-state conditions can be achieved using standardised batch cultivation [2].

We did not attempt mathematical flux modelling in this study. Instead, we took a comparative phenotyping approach aimed at the discovery of differential metabolic aspects of carbon partitioning within primary metabolism. We screened for aspects that are relevant for comparisons between HC and LC acclimation or comparisons between the chosen mutant genotypes and the *Synechocystis* WT strain. For this purpose, we combined metabolic flux and pool size assessments as previously suggested and applied [4,5]. We profiled the pool size changes of selected metabolites as our phenotype readouts. Exact quantification was performed by normalisation to the amount of chlorophyll *a* (Chl), which was invariant under our experimental conditions. These conventional metabolomic data were extended by parameters derived from mass isotopomer distribution analyses, i.e., the initial rate of ^13^C accumulation during the first 10 min and the maximum ^13^C fractional enrichment at 20-60 min. Both parameters were taken from the transient labelling kinetics of each of the monitored metabolite pools (e.g. Fig. 1).

Validation of the experimental design

By analysis of 3PGA as the main entry point of the ^13^C-label in photoautotrophic cultures of *Synechocystis*, we estimated the general agreement of the chosen phenotyping design with previous reports. After long-term (24 h) pre-acclimation to HC and LC and throughout the VHC pulse, 3PGA levels were similar and clearly increased upon initiation of both the HC and LC chase phases (Fig. 1A). As both chase conditions represent a shift from very high to relatively low inorganic carbon availability, these observations were in agreement with the increase in the 3PGA pool that was previously observed 3 h after the shift from HC to LC [2]. Similarly, the PEP concentrations were also reproducibly higher in the LC condition than in the HC condition. In the current experiment, the PEP concentration increased upon initiation of both chase regimes, with the LC condition resulting in a larger increase (Fig. 1B).

To assess the step changes during the pulse and chase, we monitored the transient ^13^C labelling of the 3PGA pool. Under our labelling conditions, the 3PGA pool reached a maximum saturated fractional enrichment 10 min after the pulse in both the HC and the LC acclimated cells. LC cells showed a more rapid ^13^C accumulation. During the chase period with the HC condition, fractional enrichment returned to below 10% within 30 min. The LC condition exhibited a delayed chase response. The empirical mass distribution vectors of 3PGA and PEP (Suppl. Fig. S1) were in general agreement with the modelling prediction of the mass isotopomer behaviour, as previously reported [4].

Our data are in agreement with the assumption that an approximately rectangular step change was applied when initiating the ^13^C-VHC pulse. The presence of ambient CO_2_, however, resulted in a perceptible isotope dilution compared to the 98% enrichment of the applied NaHCO_3_. For example, the final ^13^C enrichment in the 3PGA pool was on average >82% and >89% for HC and LC conditions, respectively (Supplementary Table S1). Moreover, the label exchange for the chase phase indicated a near-step change only for the HC condition. Under LC conditions, the label exchange was clearly delayed (Fig. 1). Dilution and carryover effects were unavoidable in our hands, especially in the case of removing the ^13^C-label upon chase initiation with LC conditions. The slow return of the ^13^C-label in 3PGA can be caused either by an insufficient physical dilution of the ^13^C-label or by a physiological effect of the CCM, which is activated under LC but suppressed under HC conditions. Therefore, in this study we propose the use of the enrichment data from the first assimilation products, e.g., 3PGA, to correct for these effects (cf. below).

Error estimation and minimisation are relevant for the judgement of phenotyping results. We need to bear in mind that the technical precision of determining the ^13^C fractional enrichment of a metabolite pool is in general below 2% relative standard deviation (RSD) when using the GC-TOF-MS metabolite profiling method [4]. In contrast, the technical error of metabolite pool size determination using the same method is about one order of magnitude greater, with the majority of measurements falling into the range of 5-20% RSD [6]. In addition to the technical error, the biological variability, i.e., the culture-to-culture differences of replicated *Synechocystis* experiments, was considered using the 3PGA measure as a test case. The precision of 3PGA pool size determinations in replicate WT and mutant cultures has previously been determined to amount to 25.6% RSD (n = 9), which is similar to the average 22.9% RSD (n = 9) of all metabolites observable by GC-TOF-MS profiling (Suppl. Table 1 of [2]). The culture-to-culture variation of the measurements introduced in this study was smaller, which was in agreement with the enhanced analytical precision of mass isotopomer distribution estimates. The maximum ^13^C fractional enrichment of the 3PGA or PEP pools determined as atom% at 20-60 min had, on average, 6.3% RSD, while the initial rate of ^13^C-accumulation in either of the pools, determined as the atom% min^-1^ by linear regression (r² = 0.85-0.99), exhibited, on average, 17.1% RSD, as assessed by triplicate WT and duplicate mutant experiments (Table 1).

**References**

1. Yang C, Hua Q, Shimizu K (2002) Metabolic flux analysis in *Synechocystis* using isotope distribution from C-13-labeled glucose. Met Engin 4: 202-216.

2. Eisenhut M, Huege J, Schwarz D, Bauwe H, Kopka J, Hagemann M (2008) Metabolome phenotyping of inorganic carbon limitation in cells of the wild type and photorespiratory mutants of the cyanobacterium *Synechocystis sp.* strain PCC 6803. Plant Physiol 148: 2109-2120.

3. Shastri AA, Morgan JA (2007) A transient isotopic labelling methodology for C-13 metabolic flux analysis of photo autotrophic microorganisms. Phytochem 68: 2302-2312.

4. Huege J, Sulpice R, Gibon Y, Lisec J, Koehl K, et al. (2007) GC-EI-TOF-MS analysis of in vivo-carbon-partitioning into soluble metabolite pools of higher plants by monitoring isotope dilution after (^13^CO_2_)-labelling. Phytochem 68: 2258-2272.

5. Zamboni N, Sauer U (2009) Novel biological insights through metabolomics and C-13-flux analysis. Current Opin Microbiol 12: 553-558.

6. Allwood JW, Erban A, de Koning S, Dunn WB, Luedemann A, et al. (2009) Inter-laboratory reproducibility of fast gas chromatography–electron impact–time of flight mass spectrometry (GC–EI–TOF/MS) based plant metabolomics. Metabolomics 5: 479–496
